# Exploring metabolomics for colorectal cancer risk prediction: evidence from the UK Biobank and ESTHER cohorts

**DOI:** 10.1186/s12916-025-04107-w

**Published:** 2025-05-13

**Authors:** Teresa Seum, Rafael Cardoso, Joshua Stevenson-Hoare, Bernd Holleczek, Ben Schöttker, Michael Hoffmeister, Hermann Brenner

**Affiliations:** 1 Division of Clinical Epidemiology and Aging Research, German Cancer Research Center (DKFZ), Im Neuenheimer Feld 581, Heidelberg, 69120 Germany; 2 Medical Faculty Heidelberg, Heidelberg University, Im Neuenheimer Feld 672, Heidelberg, 69120 Germany; 3 Saarland Cancer Registry, Präsident-Baltz-Strasse 5, Saarbrücken, 66119 Germany; 4 German Cancer Consortium (DKTK), German Cancer Research Center (DKFZ), Im Neuenheimer Feld 280, Heidelberg, Germany 69120

**Keywords:** Colorectal cancer, Metabolomics, Risk stratification, Biomarkers

## Abstract

**Background:**

While metabolic pathway alterations are linked to colorectal cancer (CRC), the predictive value of pre-diagnostic metabolomic profiling in CRC risk assessment remains to be clarified. This study evaluated the predictive performance of a metabolomics risk panel (MRP) both independently and in combination with established risk factors.

**Methods:**

We derived, internally validated (IV), and externally validated (EV) a metabolomics risk panel (MRP) for CRC from data of the UK Biobank (UKB) and the German ESTHER cohort. Baseline blood samples were assessed for 249 metabolites using nuclear magnetic resonance spectroscopy analysis. We applied LASSO Cox proportional hazards regression to identify metabolites for inclusion in the MRP and evaluated the model performance using the concordance index (C-index). We compared the performance of the MRP to an environmental risk panel (ERP; sex, age, body mass index, smoking status, and alcohol consumption) and a genetic risk panel (GRP; polygenic risk score).

**Results:**

The study included 154,892 participants of the UKB cohort (mean age at baseline 54.5 years; 55.5% female) with 1879 incident CRC and 3242 participants of the ESTHER cohort (mean age 61.5 years; 52.2% female) with 103 CRC cases. Twenty-three metabolites, primarily amino acid and lipid-related metabolites, were selected for the MRP, showing moderate predictive performance (C-index 0.60 [IV] and 0.54 [EV]). The ERP and GRP showed superior performance, with C-index values of 0.73 (IV) and 0.69 (EV). Adding the MRP to these risk models did not change the C-indices in both cohorts.

**Conclusions:**

Genetic and environmental risk information provided strong predictive accuracy for CRC risk, with no improvements from adding metabolomics data. These findings suggest that metabolomics data may have limited impact on enhancing established CRC risk models in clinical practice.

**Supplementary Information:**

The online version contains supplementary material available at 10.1186/s12916-025-04107-w.

## Background

Colorectal cancer (CRC) ranks as the second leading cause of global cancer-related deaths, accounting for 1.9 million new cases and 904,000 deaths in 2022 [1]. Its gradual progression, often lacking noticeable symptoms in its early stages, significantly contributes to its high mortality rate, largely due to delayed detection [2]. Timely screening allows for early identification and removal of precancerous lesions eventually leading to a reduction of a substantial portion of these deaths [3]. Consequently, there is major interest in identifying reliable biomarkers that facilitate early detection and risk stratification.

In recent years, metabolomics, which entails the comprehensive analysis of small molecule metabolites within biological systems, has emerged as a promising way for identifying biomarkers for various diseases [4]. Metabolomics profiling enables the quantification of numerous biomarkers across diverse biological pathways, influenced by genetic variations and environmental exposures, such as diet and smoking, in a single comprehensive measurement [5]. In the context of CRC, key pathways such as lipid metabolism, inflammation, and microbial metabolism are thought to play critical roles in tumor development and progression, providing a mechanistic basis for incorporating metabolomics into CRC risk prediction [6].

Despite systematic reviews supporting the utility of metabolomics for early CRC detection, existing studies were predominantly conducted in clinical settings, utilizing samples from diagnosed cases [6, 7]. Few studies have explored biomarkers in pre-diagnostic settings such as screening or prospective cohorts [8]. Moreover, the lack of standardization in procedures and biospecimen selection and the lack of external validation of promising results underscore the need for further work before establishing a standard clinical metabolomics biomarker panel for CRC early detection or risk stratification [6, 7].

This study employed a rigorous protocol including discovery, internal, and external validation to derive and validate a metabolomics risk panel, based on 249 metabolite biomarkers obtained through high-throughput nuclear magnetic resonance techniques, for CRC risk prediction in two large prospective cohorts of older adults, the UK Biobank and the German ESTHER cohort. We compared the effectiveness of metabolomics in CRC risk prediction with established panels for CRC risk prediction, a panel of confirmed environmental risk factors and a polygenic risk score [9].

## Methods

### The UK Biobank cohort

The UK Biobank (UKB) study is a population-based cohort study with over half a million adults recruited at ages 40–69 years across 22 assessment centers in England, Scotland, and Wales. Detailed study protocols are available on the UKB website (https://www.ukbiobank.ac.uk/). In brief, during the baseline recruitment visit between 2006 and 2010, biological samples (blood, stool, and urine) were collected in addition to information on sociodemographic, health and medical history, anthropometric, and lifestyle factors. Follow-up of health-related outcomes was conducted through linkage to electronic health records, including death and cancer from the UK National Health Service (for more details see Additional file 1: Additional Methods).

### The ESTHER cohort

The ESTHER study (German full name: Epidemiologische Studie zu Chancen der Verhütung, Früherkennung und optimierten Therapie chronischer Erkrankungen in der älteren Bevölkerung) is a statewide population-based prospective cohort study conducted in Saarland, Germany. Details of the ESTHER study have been published elsewhere [10]. In brief, 9949 women and men aged 50–75 years were recruited between 2000 and 2002 by general practitioners (GPs) during a routine health at baseline, sociodemographic characteristics, lifestyle factors, and health-related information was obtained by standardized self-administered participant and GPs questionnaires, and biological samples (blood, stool, and urine) were collected. Follow-up was performed with respect to total and cause-specific mortality, repeat questionnaires to participants and GPs, and record linkage with the Saarland Cancer Registry with respect to cancer incidence (see Additional file 1: Additional Methods).

### Data ascertainment and laboratory measurements

Demographic and lifestyle information was ascertained at baseline through self-administered questionnaires and/or physician reports. Laboratory measurements, i.e., metabolomics analysis and genotyping, were conducted on blood samples which were obtained at baseline and have been previously described [11–16]. Details regarding data ascertainment and laboratory measurements can be found in Additional file 1: Additional Methods.

### Statistical analysis

**Fig. 1:**
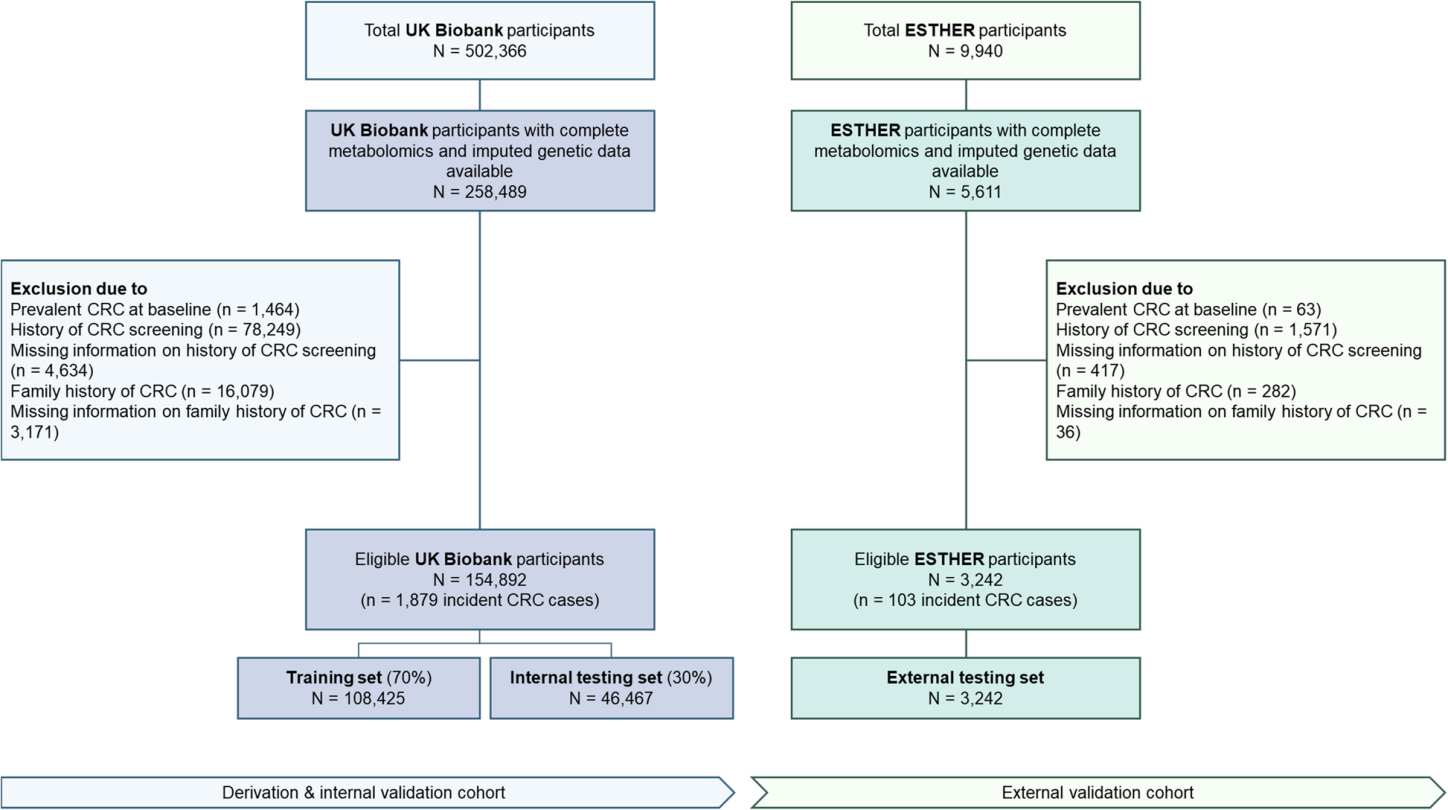
Flow diagram showing selection of study participants from the UK Biobank and the ESTHER cohorts. CRC, colorectal cancer

For both cohorts, only participants with metabolomics and genetic measurements were included. Furthermore, participants with prevalent CRC at baseline, with a history of CRC or bowel cancer screening, and those with a family history of CRC (defined as father, mother, or siblings ever diagnosed with CRC) were excluded (Fig. 1) to ensure the study represented an average-risk population for CRC screening.

Cohort characteristics were summarized by descriptive statistics. Continuous variables were described with mean (standard deviation, SD), and categorical variables were described with numbers and percentage. The values of all metabolites were log1p-transformed followed by a standardization for the following analyses.

**Fig. 2:**
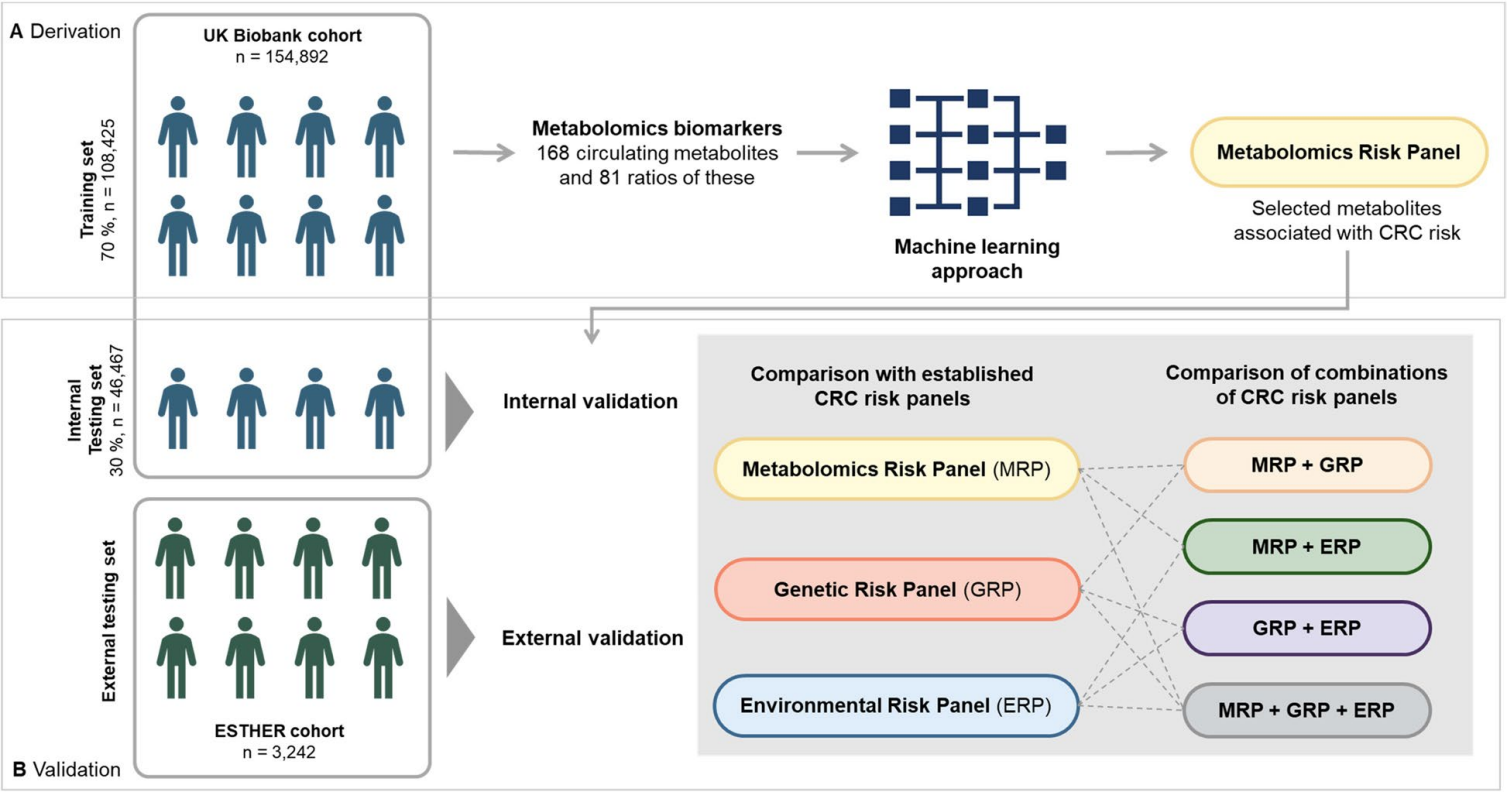
Data processing and analyses flow diagram. The figure summarizes the development and validation of the metabolomics risk panel (MRP). Participants from the UK Biobank were split into a training set (70%) and a testing set (30%). In the training set, LASSO Cox regression identified predictive metabolites, and the MRP was validated in the testing set and the ESTHER cohort. Predictive performance was assessed using C-index and compared to the genetic risk panel (GRP) and environmental risk panel (ERP), individually and in combination. CRC, colorectal cancer

The flow diagram of the analyses is shown in Fig. 2. For the main analysis, the UKB cohort participants were randomly split into a training set (70%) and a testing set (30%) for the derivation and internal validation of the metabolomics risk panel (MRP), respectively. In the discovery phase, the UKB training set was used to select the metabolites for the MRP employing a least absolute shrinkage and selection operator (LASSO) Cox proportional hazards (PH) regression model. This model, which facilitates the analysis of correlated variables, applied an L1-norm penalty to reduce the absolute values of the beta coefficients, setting those below a specified threshold (lambda) to zero. The optimal lambda was identified via tenfold cross-validation. Metabolites consistently selected in ≥ 95% of 100 iterations were included in the final MRP. To assess multicollinearity between the selected metabolites, we calculated variance inflation factors (VIF) and Pearson correlation coefficients in the training set. A VIF above 5 or a Pearson correlation coefficient higher than 0.8 indicated high multicollinearity among metabolites.

Cause-specific Cox PH models were employed to assess the association between the selected metabolites and CRC risk. The time at study entry was defined as the age at recruitment, while exit time was determined by the age at incident CRC diagnosis, death, or the last date at which follow-up was considered complete. Hazard ratios (HR) with 95% confidence intervals (CI) were calculated per one standard deviation (SD) increase of the log1p-transformed value for each individual selected metabolite of the MRP. Multiple testing corrections were applied using false-discovery rate (FDR) correction [17].

In the validation phase, the MRP was internally validated using the UKB testing set and externally validated within the ESTHER cohort. Performance comparisons for CRC risk prediction were made between the MRP, a genetic risk panel (GRP) based on a polygenic risk score, and an environmental risk model (ERP) incorporating factors such as age, sex, body mass index (BMI), smoking status, and alcohol consumption, selected based on prior evidence [18]. Additionally, combinations of these models (MRP + GRP, MRP + ERP, GRP + ERP, and MRP + GRP + ERP) were evaluated. The predictive accuracy of each model was assessed using concordance index (C-index), and the stability and precision of the C-indices were further evaluated through bootstrap resampling of 1000 samples to calculate 95% CI. Furthermore, we repeated the analysis by only including 5-year and 10-year follow-up periods to analyze the model performance over these specific time frames.

### Sensitivity analyses

To determine whether incorporating the selected metabolites of the MRP improved existing risk prediction panels (GRP and ERP), we evaluated the net reclassification index (NRI) and the integrated discrimination improvement (IDI), when adding the MRP to the GRP and the ERP individually, as well as to a combined GRP and ERP model. Additionally, we examined the discriminatory ability of the MRP within subgroups of the ERP by calculating the C-index separately for sex (female, male), age (< 60 years, ≥ 60 years), BMI (< 25, 25–30, ≥ 30 kg/m^2^), smoking status (never, former, current), and alcohol consumption (abstainer/low, moderate/ high).

To assess whether the association between MRP and CRC risk differed across these subgroups, we performed stratified Cox regression analyses to estimate HRs within each subgroup. Furthermore, interaction terms (MRP × age, MRP × sex, MRP × BMI, MRP × smoking status, MRP × alcohol consumption) were incorporated into Cox regression models to test for effect modification.

Analyses were conducted using R statistical software, version 4.3.1. Tests for statistical significance were two-sided with an alpha value of 0.05.

## Results

### Baseline characteristics of the study participants

A total of 154,892 eligible participants from the UKB cohort and 3242 participants from the ESTHER cohort were included in this analysis (Fig. 1). The baseline characteristics of the study population are summarized in Table 1, stratified by cohort. Additionally, the distribution of the training and testing sets of the UKB cohort is described in Additional file 2: Table S2. Participants in the ESTHER cohort were slightly older than those in the UKB cohort (mean ages 61.5 and 54.5, respectively). Over a median follow-up period of 12.1 years, 1879 CRC cases were observed in the UKB cohort. In the ESTHER cohort, 103 CRC cases occurred over a median follow-up period of 17.5 years. Median time from baseline to diagnosis was 6.9 years for CRC cases in the UKB cohort and 7.6 years for CRC cases in the ESTHER cohort. Notably, both cohorts included more women than men, but the majority of cases were men (55.3% in UKB, 66.0% in ESTHER). CRC cases in both cohorts furthermore had a higher mean BMI, more often smokers, and consumed more alcohol.

**Table 1:** Baseline characteristics of the study participants, by cohort

Baseline characteristics	UK Biobank cohort				ESTHER cohort			
	Participants (*n* = 154,892)	Cases (*n* = 1879)	Non-cases (*n* = 153,070)	*p* value	Participants (*n* = 3242)	Cases (*n* = 103)	Non-cases (*n* = 3139)	*p* value
Sex, No. (%)				< 0.001				< 0.001
Male	68,952 (44.5)	1040 (55.3)	67,912 (44.4)		1551 (47.8)	68 (66.0)	1483 (47.2)	
Female	85,940 (55.5)	839 (44.7)	85,101 (55.6)		1691 (52.2)	35 (34.0)	1656 (52.8)	
Age at blood collection, in years				< 0.001				< 0.001
Mean (SD)	54.5 (7.98)	59.12 (7.20)	54.45 (7.97)		61.5 (6.62)	63.65 (6.04)	61.42 (6.63)	
BMI (kg/m^2^)				< 0.001				0.024
Mean (SD)	27.35 (4.82)	28.04 (4.95)	27.35 (4.81)		27.68 (4.27)	28.63 (4.24)	27.65 (4.27)	
Unknown	567	4	563		4	–	4	
Smoking status, No. (%)				< 0.001				0.015
Never	88,045 (56.8)	887 (47.2)	87,158 (57.0)		1558 (48.1)	44 (42.7)	1514 (48.2)	
Former	49,639 (32.1)	776 (41.3)	48,863 (31.9)		1068 (32.9)	47 (45.6)	1021 (32.5)	
Current	17,163 (11.1)	216 (11.5)	16,947 (11.1)		556 (17.2)	11 (10.7)	545 (17.4)	
Unknown	45	–	45		60	1	59	
Alcohol consumption, No. (%)				< 0.001				0.313
Abstainer	48,084 (31.0)	517 (27.5)	47,567 (31.1)		1180 (28.9)	25 (22.6)	890 (29.1)	
Low	61,727 (39.9)	760 (40.4)	60,967 (39.9)		2308 (56.5)	68 (65.3)	1796 (56.2)	
Medium	26,554 (17.1)	330 (17.6)	26,224 (17.1)		199 (4.8)	4 (4.0)	155 (4.9)	
High	18,527 (12.0)	272 (14.5)	18,255 (11.9)		63 (1.5)	3 (3.2)	41 (1.5)	
Unknown	–	–	–		260	3	257	
Follow-up time to CRC diagnosis, in years				–				–
Median (range)	–	6.85 (0–15)	–		–	7.63 (0–18)	–	

*BMI* Body mass index, *CRC* Colorectal cancer, *No* Number, *SD* Standard deviation

*p *values were obtained from t-test for continuous variables and from chi-squared test for categorical variables

### Construction of the metabolite-based risk panel

In the construction of the MRP, 25 metabolites (mainly subclasses of amino acids, glycolysis-related metabolites, ketone bodies, and relative lipoprotein lipid concentrations) were selected as components of the MRP through LASSO. To ensure the robustness of the MRP and minimize redundancy among selected metabolites, we assessed multicollinearity using VIF analysis and Pearson correlation in the UKB training set. The total concentration of branched-chain amino acids (VIF = 21.6) and valine (VIF = 21.9) exhibited the strongest multicollinearity. This was followed by the cholesteryl esters to total lipids ratio in very small very low-density lipoproteins (VLDL) (VIF = 18.9) and the triglycerides to total lipids ratio in very small VLDL (VIF = 15.9). Pearson correlation matrix (Additional file 2: Fig. S1) showed similar patterns, with strong correlations between the total concentration of branched-chain amino acids and valine (*r* = 0.97), as well as between the triglycerides to total lipids ratio and the cholesteryl esters to total lipids ratio in very small VLDL (*r* = − 0.95).

To address multicollinearity, the metabolites with the highest VIF from each correlated pair were excluded, i.e., valine and the cholesteryl esters to total lipids ratio in very small VLDL, resulting in a final MRP of 23 metabolites. After exclusion, all VIF values were < 5, and no strong correlations remained.

**Fig. 3:**
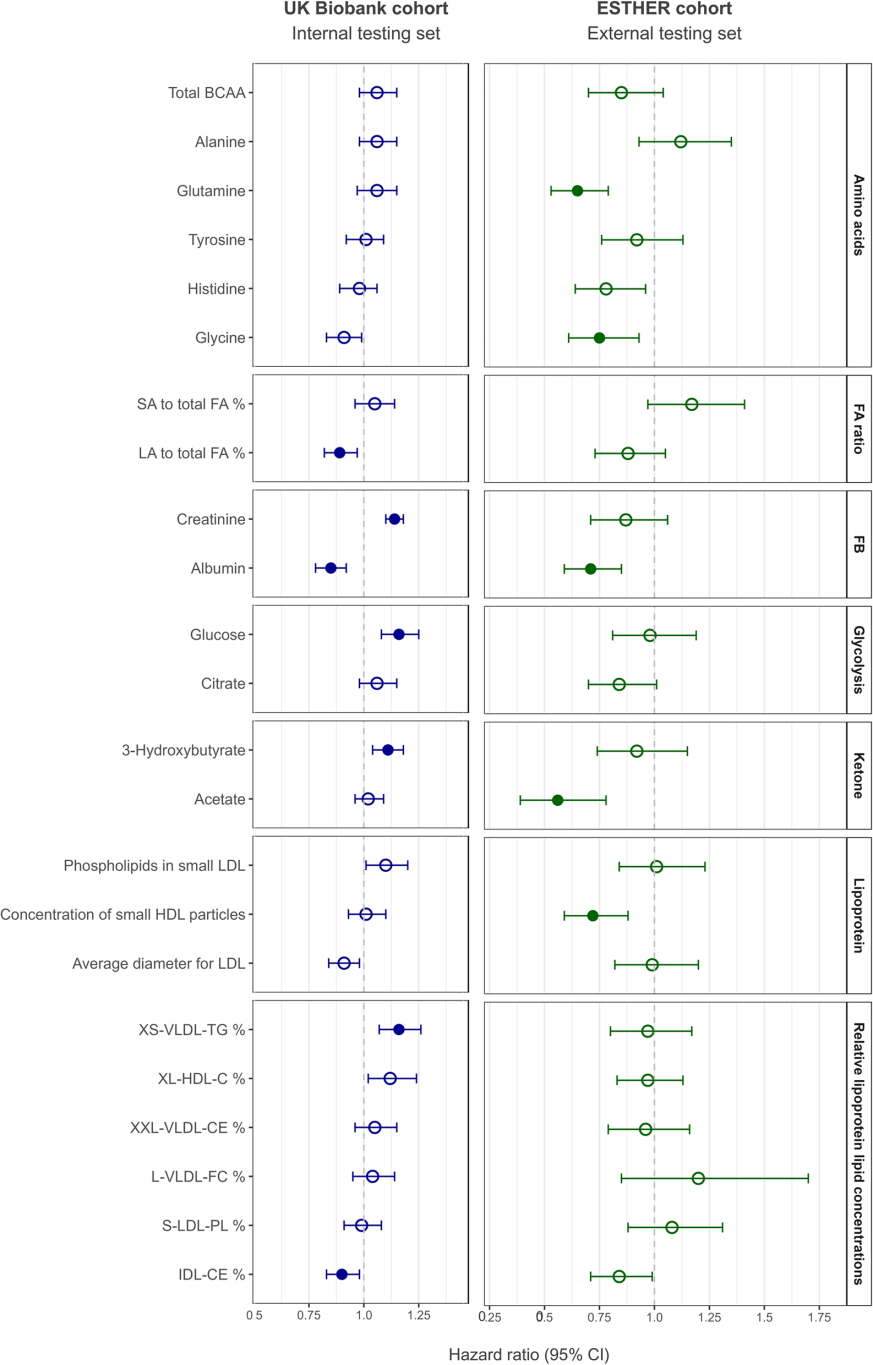
Hazard ratios (95% CI) for the selected metabolites, by cohort. Cause-specific Cox proportional hazards models were used to estimate hazard ratios (HR) and 95% confidence intervals (CI) for the association between selected metabolites and CRC risk. Age at recruitment was defined as study entry, and exit time was determined by CRC diagnosis, death, or end of follow-up. HRs were reported per 1-SD increase of the log1p-transformed value for each metabolite. Multiple testing correction was applied using the Benjamini–Hochberg method. Full dots indicate metabolites that remained significant after correction, while hollow dots indicate non-significant associations. BCAA branched-chain amino acids, C cholesterol, CE cholesteryl esters, CI confidence interval, FA fatty acids, FB fluid balance, FC free cholesterol, Glycolysis glycolysis-related metabolites, HDL high-density lipoproteins, HR hazard ratio, Ketone ketone bodies, L large, LA linoleic acid, LDL low-density lipoproteins, M medium, PL phospholipids, S small, SD standard deviation, TG triglycerides, UKB UK Biobank, VLDL very low-density lipoproteins, XL very large, XS very small, XXL extremely large, % ratio

The HRs of the selected metabolites included in the MRP are shown in Fig. 3 and Additional file 2: Table S3. After adjusting for multiple testing, in the UKB testing set, three metabolites were significantly inversely associated with CRC, while four metabolites showed a significant positive association. In the ESTHER cohort, five metabolites displayed a significant inverse association with CRC. The directionality of the significant associations in the UKB and ESTHER cohort was the same in both studies.

### Internal and external validation of metabolomics risk panel and comparison

Table 2 depicts the results of the internal and external validation of the risk panels as well as their combinations. The internal validation of the MRP yielded a C-index of 0.602 (95% CI, 0.577, 0.625). External validation in the German ESTHER cohort resulted in a C-index of 0.542 (95% CI, 0.481, 0.605).

**Table 2:** Performance of the individual and combined risk prediction panels, by internal and external validation

	Internal validation (UKB testing set, *n* = 46,467)	External validation (ESTHER cohort, *n* = 3242)
	C-index (95% CI)	
Individual panels		
Metabolomics risk panel (MRP)	0.602 (0.577, 0.625)	0.542 (0.481, 0.605)
Environmental risk panel (ERP)	0.688 (0.664, 0.710)	0.638 (0.587, 0.688)
Genetic risk panel (GRP)	0.633 (0.608, 0.655)	0.636 (0.578, 0.687)
Combined panels		
MRP + GRP	0.665 (0.641, 0.685)	0.633 (0.576, 0.689)
MRP + ERP	0.688 (0.665, 0.710)	0.647 (0.596, 0.695)
GRP + ERP	0.725 (0.703, 0.746)	0.688 (0.638, 0.734)
MRP + GRP + ERP	0.726 (0.704, 0.746)	0.692 (0.641, 0.738)

Model performance was assessed using the concordance index (C-index), with 95% confidence intervals obtained through bootstrap resampling (1000 iterations). The UKB training set was used to develop the models, which were internally validated in the UKB testing set and externally validated in the ESTHER cohort

*CI* Confidence interval, *UKB* UK Biobank

The internal validation of the ERP (including age, sex, BMI, smoking status, and alcohol consumption) yielded a C-index of 0.688 (95% CI, 0.664, 0.710). External validation in the ESTHER cohort resulted in a C-index of 0.638 (95% CI, 0.587, 0.688). The GRP yielded a C-index of 0.633 (95% CI, 0.608, 0.655) for the internal validation set and a C-index of 0.636 (95% CI, 0.578, 0.687) in the external validation set.

In a further analysis, four combined panels were created by adding up the information of the three individual panels. The combination of MRP and GRP yielded the lowest of the combined panels, with a C-index in the internal validation cohort of 0.665 (95% CI, 0.641, 0.685) and a C-index of 0.633 (95% CI, 0.576, 0.689) in the external validation cohort. The combination of MRP and ERP yielded a C-index in the internal validation cohort of 0.688 (95% CI, 0.665, 0.710) and a C-index of 0.647 (95% CI, 0.596, 0.695) in the external validation cohort. Both the combination of GRP and ERP and the combination of all panels (MRP, GRP, and ERP) yielded similar C-indices of 0.725 (95% CI, 0.703, 0.746) and 0.726 (95% CI, 0.704, 0.746) in the internal validation cohort. In the external validation cohort, the combination of all tree panels had a slightly higher C-index of 0.692 (95% CI, 0.641, 0.738) than the C-index of the GRP + ERP model (0.688 [95% CI, 0.638, 0.734]).

The results of the analysis by 5-year and 10-year follow-up are presented in Additional file 2: Table S4. For the internal validation, when only including 5-year and 10-year follow-ups, C-indices were slightly lower, the shorter the follow-up time, with a C-index of 0.590 (95% CI, 0.551, 0.624) and 0.603 (95% CI, 0.578, 0.630) for MRP in the 5-year and 10-year follow-up analyses, respectively. Of all models, the model incorporating GRP and ERP showed the highest predictive accuracy with a C-index of 0.742 (95% CI, 0.705, 0.777) over 5 years and 0.727 (95% CI, 0.703, 0.749) over 10 years in internal validation. In the external validation, the most comprehensive model, including MRP, GRP, and ERP, achieved the highest C-index of 0.739 (95% CI, 0.654, 0.820) for 5-year and 0.746 (95% CI, 0.658, 0.771) for 10-year prediction.

### Comparative analysis of model performance

We assessed the predictive performance of different models by evaluating the NRI and IDI, analyzing the impact of adding the MRP to the GRP, the ERP, and their combination (Additional file 2: Table S5). In the internal validation, adding MRP to GRP significantly improved overall reclassification (NRI: 0.189, 95% CI: 0.120 to 0.258) and discrimination (IDI: 0.045, 95% CI: 0.036, 0.053), driven mainly by better classification for non-events (NRI for non-events: 0.764, 95% CI: 0.758, 0.770). Adding MRP to ERP showed high improvement for events (NRI: 0.992, 95% CI: 0.982, 1.003) but a worse performance for non-events (NRI: − 0.998, 95% CI, − 0.999, − 0.997). Combining GRP, MRP, and ERP yielded no significant reclassification improvement in comparison with GRP + ERP (NRI: − 0.005, 95% CI, − 0.015 to 0.005).

For external validation, adding MRP to GRP showed no reclassification improvement (NRI: 0.022, 95% CI, − 0.099, 0.144) but a significant change in discrimination (IDI: 0.021, 95% CI, 0.002, 0.040). In addition, the combined model of all 3 panels demonstrated no overall improvement in reclassification (NRI: − 0.009, 95% CI, − 0.047, 0.029) over GRP + ERP but a slight improvement in discrimination (IDI: 0.004, 95% CI, 0.002, 0.006).

### Sensitivity analysis

Stratified analyses by ERP risk factors showed consistent discriminatory ability of MRP across subgroups in the internal validation cohort (Additional file 2: Table S6). Differences observed in the ESTHER cohort were accompanied by small case numbers, limiting interpretability (e.g., C-index for alcohol consumption: abstainer/low 0.516 [95% CI, 0.450, 0.576], 96 CRC cases; medium/high 0.721 [95% CI, 0.523, 0.895], 7 CRC cases).

Further examining potential effect modification, Cox regression analyses showed a positive association between MRP and CRC risk across all ERP-defined subgroups in the UKB testing set, with a significant interaction for alcohol consumption (interaction *p* < 0.05) (Additional file 2: Table S7). The association was stronger in the medium/high consumption group (HR 1.73 [95% CI, 1.39–2.17]) than in the abstainer/low group (HR 1.26 [95% CI, 1.17–1.36]). In the ESTHER cohort, no statistically significant associations or interaction effects were detected.

## Discussion

Colorectal cancer stands as a major global health challenge, requiring innovative strategies for early detection and risk prediction. While previous studies have identified metabolic pathway alterations associated with CRC, the translation into validated biomarkers panels has been scarce [7]. This study draws upon data from the UKB and the German ESTHER cohort to study the associations of metabolites with incident CRC and derive a prediction panel consisting of selected metabolites. We generated a metabolomics risk panel consisting of 23 metabolites associated with CRC risk using a LASSO Cox PH regression approach. This panel was validated internally within the UKB and externally in the ESTHER cohort. The individual performance of the metabolomics risk panel was inferior to the panels that incorporated conventional risk factors such as sex, age, BMI, smoking status, and alcohol consumption, or genetic information through polygenic risk scores. Especially the environmental risk panel showed a particularly strong performance, underscoring the relevance of established risk factors in predicting CRC risk. The combination of the environmental and genetic risk panels achieved moderate discriminatory accuracy, with C-index values exceeding 0.7 in the internal validation. Adding metabolomics to the combination of the environmental and genetic risk panels did not resulted in an improvement in performance. This indicates that a multi-dimensional approach that includes environmental and genetic data may offer the best strategy for CRC risk prediction.

There is a limited number of studies using pre-diagnostic metabolite panels for the prediction of CRC risk. Loftfield et al. conducted a study in a US-based cohort with a 10-year interval between blood draw and CRC diagnosis, focusing on short-chain fatty acids and bile acids [19]. They reported odds ratios (95% CIs) of 0.55 (0.31–0.98) for a panel consisting of six short-chain fatty acids and 1.95 (1.04–3.66) for a panel of 15 bile acids when comparing the highest compared with lowest quartile. However, this study lacked external validation, and the associations were observed for female participants only. A study in Asia of 250 incident CRC cases showed a moderate discriminatory accuracy (AUC = 0.76) for a panel of nine metabolites [20, 21], higher than in our study, though its small sample size may affect the findings’ robustness. The intriguing results of this study from Asia are yet to be confirmed by external validation in independent cohorts. In particular, further research is needed if and to what extent the proposed signatures may enhance risk prediction beyond risk prediction by established CRC risk factors.

A limited number of studies have also compared metabolite-based models to existing risk panels for CRC. The Nightingale Health Biobank Collaborative Group conducted the only known study comparing a polygenic risk score to a metabolite-based model, finding lower hazard ratios for metabolomics high-risk groups than genetic high-risk groups for CRC [22]. The only study comparing a lifestyle-based model to a metabolite-based model was performed by Rothwell et al. [23]. However, opposite to our environmental risk score, they examined the positive effects of healthy lifestyle behaviors, such as weight maintenance and physical activity, as recommended by the World Cancer Research Fund/American Institute for Cancer Research guidelines. They showed that signatures of fatty acids and endogenous metabolites had a stronger association with CRC risk than the adherence to these health-promoting guidelines [23]. These promising results also still require external validation in independent cohorts to confirm their generalizability.

Although metabolomics alone may not currently surpass the predictive accuracy of genetic and environmental factors for CRC risk, this approach still holds significant value. The panel of pre-diagnostic metabolites, even if less predictive, shows that metabolic pathways may be linked to CRC development. These insights could be crucial for future research into disease mechanisms and therapeutic targets. Metabolites associated with CRC risk, including glucose and amino acids such as glycine, alanine, tyrosine, and glutamine, are all key players in cellular energy metabolism and proliferation, fueling cancer cell proliferation and growth through metabolic reprogramming [24, 25]. Additionally, lipids such as cholesterol, triglycerides, and phospholipids, represented as relative lipoprotein lipid concentrations in the panel, also have been implicated in CRC development and progression [26, 27]. These insights offer a deeper understanding of the metabolic pathways of cancer [28].

### Strengths and limitations

This study represents one of the largest CRC metabolomics investigations to date, incorporating a substantial number of CRC cases and non-cases from two population-based cohorts. The prospective design, featuring samples collected at various time points preceding case diagnosis, adds robustness to the study’s findings. The comprehensive validation approach, encompassing both internal validation within the UKB cohort and external validation in the ESTHER cohort, ensures the reliability of our findings within the same population, and external validation ensuring the generalizability to our results. This dual-validation strategy addresses concerns about overfitting and enhances the reliability of our conclusions across diverse populations. Moreover, the application of Nuclear Magnetic Resonance (NMR) spectroscopy, a minimally invasive and high-throughput method for identifying metabolomics biomarkers, contributes to the methodological rigor of the investigation. The use of rigorous statistical methods, like the LASSO Cox proportional hazard regression model in comparison to conventional Cox models, further supports the certainty of the evidence presented.

While this study significantly advances our understanding of the pre-diagnostic metabolomics panels linked to CRC, it is essential to acknowledge certain limitations. The Nightingale metabolomics panel, based on the NMR profiling algorithm from a single company, includes a limited selection of identified metabolites, constraining the comprehensiveness of our findings. Sensitivity of metabolomics to pre-analytical sample management, influenced by factors such as freeze–thaw cycles and storage conditions, represents a noteworthy limitation [29]. However, the stringent protocols followed by the UKB cohort and the ESTHER cohort help mitigate some of these concerns. The reliance on self-reported data for lifestyle-related factors and minor changes in the questionnaire over time introduce additional limitations. Furthermore, the predominantly white European ethnicity, higher socioeconomic status of participants, and the generally healthier profile of the UKB participants may restrict the generalizability, while the age of the ESTHER cohort could limit insights into early-onset CRC risk.

## Conclusions

Despite the potential merits of metabolomics studies in pointing to potentially relevant etiological or pathomechanistic pathways, the contribution of metabolomics panels for enhanced risk stratification remains limited. Our findings, based on a robust internal and external validation framework, provide insights into the relationship between metabolites and CRC risk. However, the inclusion of metabolites alongside environmental or genetic risk panels did not lead to substantial improvement in predictive accuracy. This suggests that while metabolomics may offer some insights, clinical relevance in CRC risk assessment requires further investigation. Further research potentially including more specific and precise metabolomics phenotyping, along with complementary and more comprehensive -omics approaches in large cohorts, including rigorous internal and external validation, may be needed to unravel clinically relevant contributions of metabolomics in CRC risk assessment.

## Supplementary Information

Additional file 1: Additional Methods. Data ascertainment and laboratory measurements.

Additional file 2: Table S1 Overview on colorectal cancer–related single-nucleotide variants identified in genome-wide association studies and considered in this analysis, based on Thomas et al. Table S2 Baseline characteristics of study participants stratified by training or testing set in the UK Biobank cohort. Table S3 *β* coefficient of HR (95% CI) and *p* values of colorectal cancer for the selected metabolites in Cox proportional hazards model. Table S4 Performance of the individual and combined 5-year and 10-year risk prediction panels, by internal and external validation. Table S5 Diagnostic performance of NRI and IDI between MRP and the combined models. Table S6 Discriminatory ability of the MRP in subgroups defined by ERP risk factors. Table S7 Cox proportional hazards models of the MRP in subgroups defined by ERP risk factors. Fig. S1 Correlation matrix of the selected metabolites in the UK Biobank training set.

## Data Availability

ESTHER: Due to restrictions of informed consent, the data cannot be made publicly available, but they are available upon reasonable request with appropriate proposal and approval from the ESTHER principal investigator. UK Biobank: We have received access to and permission to use these data upon request from the UK Biobank. We are not allowed to pass these data to others. However, researchers aiming to use the dataset our analysis is based on can obtain access to and permission to use the data from the UK Biobank upon their own request to the UK Biobank.

## Abbreviations

BMI: Body mass index
CI: Confidence intervals
C-index: Concordance index
CRC: Colorectal cancer
ERP: Environmental risk model
ESTHER: Epidemiologische Studie zu Chancen der Verhütung, Früherkennung und optimierten Therapie chronischer Erkrankungen in der älteren Bevölkerung [German name]
FDR: False-discovery rate
GP: General practitioner
GRP: Genetic risk panel
IDI: Integrated discrimination improvement
LASSO: Least absolute shrinkage and selection operator
MRP: Metabolomics risk panel
NMR: Nuclear magnetic resonance
No: Number
NRI: Net reclassification index
PH: Proportional hazards
SD: Standard deviation
VIF: Variance inflation factor
UKB: UK Biobank
