# Supplementary material for: Exploring metabolomics for colorectal cancer risk prediction: evidence from the UK Biobank and ESTHER cohorts
Source: BMC Med. 2025 May 13;23:283. doi: 10.1186/s12916-025-04107-w (PMC12077020; doi:10.1186/s12916-025-04107-w)
Supplement: Supplementary file 1 — Additional file 1: Additional Methods. Data ascertainment and laboratory measurements. [file 12916_2025_4107_MOESM1_ESM.docx]

**Additional File 1**

**Additional Methods**. Data ascertainment and laboratory measurements

**Additional Methods. Data ascertainment and laboratory measurements**

**Metabolomics**

Detailed protocols on sample collection for the UKB cohort are presented elsewhere [1-3]. Briefly, EDTA plasma samples were collected during the baseline assessment of the UKB and underwent analysis in two phases spanning 2019-2022. Serum samples from the participants in the ESTHER cohort were obtained at recruitment and stored at -80°C until analysis which took place in 2022 and 2023.

Targeted high-throughput nuclear magnetic resonance (NMR) metabolomics by Nightingale Health Ltd (biomarker quantification version 2020) was employed for the analysis of subsets from both cohorts. The UKB subset comprised 274,353 randomly selected baseline plasma samples, while the ESTHER subset included all 8,308 available baseline serum samples with sufficient blood sample quality. This methodology enabled the simultaneous quantification of 249 metabolic measures, encompassing routine lipids, lipoprotein subclass profiling, fatty acid composition, and various low-molecular-weight metabolites, such as amino acids, ketone bodies, and glycolysis metabolites, all quantified in molar concentration units. Stringent quality control measures were consistently applied throughout the process to eliminate systemic and technical variance. Only samples and biomarkers that successfully passed the quality control process were retained in the respective datasets and employed in the current study.

**Environmental risk factors**

Information on environmental risk factors (established risk factors for CRC), including age, sex, body mass index (BMI), smoking status, and alcohol consumption were extracted from participants’ questionnaires administered at baseline in both cohorts. Age (UKB Field ID: 21003) and BMI (UKB Field ID: 21001) were continuous variables and sex (male/female, UKB Field ID: 31), and smoking status (never, former, current; UKB Field ID: 1239 for current smoking and 1249 for past smoking) were defined as categorical variables. Alcohol consumption was based on the weekly intake of red wine (UKB Field ID: 1568), champagne/white wine (1578), beer/cider (1588), spirits (1598), and fortified wine (1608) in the UK Biobank cohort, and as weekly intake of wine, beer, and liquor in the ESTHER cohort. It was categorized as Abstainer; low = females 0–19.99 g/day or males 0–39.99 g/day; medium = females 20–39.99 g/day or males 40–59.99 g/day; high = females ≥40 g/day or males ≥60 g/day.

Missing values for the risk factors were replaced by imputation using the R Package missForest (UK Biobank cohort: BMI n = 567, smoking status n = 45*;* ESTHER cohort: BMI n = 4, smoking status n = 60, and alcohol consumption n = 160) [4].

**Derivation of polygenic risk scores**

Genotyping, quality control, and imputation details for the UK Biobank cohort have been previously described [5]. Briefly, genotyping involved the use of the UK BiLEVE Axiom and the UK Biobank Axiom arrays for approximately 50,000 and 450,000 participants, respectively, both sharing 95% of their marker content. Imputation was conducted using either the Haplotype Reference Consortium or a combined reference panel from the 1000 Genomes Project and UK10K. Additionally, Thompson et al. [6] developed a standard polygenic risk score (PRS) Set for 28 diseases and eight quantitative traits by conducting a meta-analysis of various external GWAS sources. This set employs genome-wide variant-derived PRSs calculated using a Bayesian approach. In our study, we focused on the standard PRS for colorectal cancer.

For the ESTHER cohort, extracted DNA from blood cell collected at baseline from available serum samples with sufficient blood sample quality was genotyped using the Illumina OncoArray BeadChip (for 82% of the participants) and Global Screening Array (for 18% of the participants). Quality control of the genotype data was performed following a standardized protocol. Missing genotypes (~40 million SNPs) were imputed using Haplotype Reference Consortium (version r1.1.2016) as reference panel within the Michigan Imputation Server. PLINK (version 1.9) was used to extract SNPs for the required region of interest. The PRS for CRC was built based on 139 of 140 CRC-related risk variants that were identified in a recent genome-wide association study of CRC risk within individuals of European ancestry (Additional File 2: **Table S1**; rs6928864 was not measured and thus was not included in the analysis) [7]. The PRS for each participant was calculated as a weighted sum of risk alleles using weights reported by Thomas et al. [7].

**Colorectal cancer outcomes**

The identification of CRC cases in both cohorts relied on the 10th Revision of the International Classification of Diseases (ICD-10). This encompassed proximal colon cancers (C18.0 and C18.2–18.5), distal colon cancers (C18.6–C18.7), overlapping and unspecified lesions of the colon (C18.8–C18.9), and rectal cancers (C19–C20). Date of complete follow-up of cancer incidence data in the UKB cohort was December 31^st^ 2020 for England, December 31^st^ 2016 for Wales, and January 31^st^ 2021 for Scotland. The censoring date for death data was November 30^th^ 2022 for the whole UK Biobank cohort. The ESTHER cohort’s date of complete follow-up for both cancer incidence and mortality data was December 31^st^ 2018.

**References**

1. Soininen P, Kangas AJ, Wurtz P, Suna T, Ala-Korpela M. Quantitative serum nuclear magnetic resonance metabolomics in cardiovascular epidemiology and genetics. Circ Cardiovasc Genet. 2015;8(1):192-206. doi: 10.1161/CIRCGENETICS.114.000216.

2. Soininen P, Kangas AJ, Wurtz P, Tukiainen T, Tynkkynen T, Laatikainen R, et al. High-throughput serum NMR metabonomics for cost-effective holistic studies on systemic metabolism. Analyst. 2009;134(9):1781-5. doi: 10.1039/b910205a.

3. Wurtz P, Kangas AJ, Soininen P, Lawlor DA, Davey Smith G, Ala-Korpela M. Quantitative Serum Nuclear Magnetic Resonance Metabolomics in Large-Scale Epidemiology: A Primer on -Omic Technologies. Am J Epidemiol. 2017;186(9):1084-96. doi: 10.1093/aje/kwx016.

4. Stekhoven DJ, Stekhoven MDJ. Package ‘missForest’. Bioinformatics. 2013;28(1):112-8. doi: 10.1093/bioinformatics/btr597.

5. Bycroft C, Freeman C, Petkova D, Band G, Elliott LT, Sharp K, et al. The UK Biobank resource with deep phenotyping and genomic data. Nature. 2018;562(7726):203-9. doi: 10.1038/s41586-018-0579-z.

6. Thompson DJ, Wells D, Selzam S, Peneva I, Moore R, Sharp K, et al. UK Biobank release and systematic evaluation of optimised polygenic risk scores for 53 diseases and quantitative traits. medRxiv. 2022. doi: 10.1101/2022.06.16.22276246.

7. Thomas M, Sakoda LC, Hoffmeister M, Rosenthal EA, Lee JK, van Duijnhoven FJB, et al. Genome-wide Modeling of Polygenic Risk Score in Colorectal Cancer Risk. Am J Hum Genet. 2020;107(3):432-44. doi: 10.1016/j.ajhg.2020.07.006.
