# Supplementary material for: Exploring metabolomics for colorectal cancer risk prediction: evidence from the UK Biobank and ESTHER cohorts
Source: BMC Med. 2025 May 13;23:283. doi: 10.1186/s12916-025-04107-w (PMC12077020; doi:10.1186/s12916-025-04107-w)
Supplement: Supplementary file 2 — Additional file 2: Table S1 Overview on colorectal cancer–related single-nucleotide variants identified in genome-wide association studies and considered in this analysis, based on Thomas et al. Table S2 Baseline characteristics of study participants stratified by training or testing set in the UK Biobank cohort. Table S3 β coefficient of HR (95% CI) and p values of colorectal cancer for the selected metabolites in Cox proportional hazards model. Table S4 Performance of the individual and combined 5-year and 10-year risk prediction panels, by internal and external validation. Table S5 Diagnostic performance of NRI and IDI between MRP and the combined models. Table S6 Discriminatory ability of the MRP in subgroups defined by ERP risk factors. Table S7 Cox proportional hazards models of the MRP in subgroups defined by ERP risk factors. Fig. S1 Correlation matrix of the selected metabolites in the UK Biobank training set. [file 12916_2025_4107_MOESM2_ESM.docx]

**Additional File 2**

**Tables**

### **Table S1**. Overview on colorectal cancer–related single-nucleotide variants identified in genome-wide association studies and considered in this analysis, based on Thomas et al.

### **Table S2**. Baseline characteristics of study participants stratified by training and testing set in the UK Biobank cohort.

# **Table S3**. β coefficient of HR (95% CI) and *p* values of colorectal cancer for the selected metabolites in cox proportional hazards model

**Table S4.** Performance of the individual and combined risk prediction panels (5-year and 10-year follow-up), by internal and external validation

**Table S5**. Diagnostic performance of NRI and IDI between MRP and the combined models

**Table S6**. Discriminatory ability of the MRP in subgroups defined by ERP risk factors

**Table S7**. Cox proportional hazards models of the MRP in subgroups defined by ERP risk factors

**Figures**

**Figure S1**. Correlation matrix of the selected metabolites in the UK Biobank training set

### **Table S1.** Overview on colorectal cancer–related single-nucleotide variants identified in genome-wide association studies and considered in this analysis, based on Thomas et al. [9]

| **SNP** | **Locus** | **Position** | **Risk allele** | **Beta** |
| --- | --- | --- | --- | --- |
| rs4360494 | 1p34.3 | 38455891 | G | 0.0379 |
| rs12144319 | 1p32.3 | 55246035 | C | 0.0661 |
| rs72647484 | 1p36.12 | 22587728 | T | 0.0504 |
| rs7542665 | 1p31.3 | 62673037 | C | 0.0334 |
| rs6678517 | 1q25.3 | 183002639 | A | 0.073 |
| rs17011141 | 1q41 | 222112634 | G | 0.0877 |
| rs448513 | 2q24.2 | 159964552 | C | 0.0054 |
| rs11884596 | 2q33.1 | 199612407 | C | 0.0342 |
| rs983402 | 2q33.1 | 199781586 | T | 0.0622 |
| rs7606562 | 2p16.3 | 48686695 | T | 0.0414 |
| rs11692435 | 2q11.2 | 98275354 | G | 0.0492 |
| rs3731861 | 2q35 | 219191256 | T | 0.0613 |
| rs10049390 | 3q22.2 | 133701119 | A | 0.0455 |
| rs13086367 | 3q13.2 | 112903888 | A | 0.0463 |
| rs72942485 | 3q13.2 | 112999560 | G | 0.0545 |
| rs9831861 | 3p21.1 | 53088285 | G | 0.0294 |
| rs35470271 | 3p22.1 | 40915239 | G | 0.0994 |
| rs12635946 | 3q13.2 | 112916918 | C | 0.0334 |
| rs113569514 | 3q22.2 | 133748789 | T | 0.0414 |
| rs9876206 | 3q26.2 | 169517436 | C | 0.0453 |
| rs6781752 | 3p14.1 | 66365163 | A | 0.0597 |
| rs11727676 | 4q31.21 | 145659064 | C | 0.0093 |
| rs1391441 | 4q24 | 106128760 | A | 0.0148 |
| rs13149359 | 4q22.2 | 94938618 | A | 0.052 |
| rs7708610 | 5p13.1 | 40102443 | A | 0.0384 |
| rs78368589 | 5p15.33 | 1240204 | T | 0.0786 |
| rs145364999 | 5q21.1 | 98206082 | T | 0.3496 |
| rs2735940 | 5p15.33 | 1296486 | G | 0.0865 |
| rs12514517 | 5p13.1 | 40280076 | A | 0.1013 |
| rs755229494 | 5q22.2 | 112097351 | G | 0.6286 |
| rs12659017 | 5q23.2 | 125988175 | G | 0.0374 |
| rs4976270 | 5q31.1 | 134467220 | C | 0.0693 |
| rs13204733 | 6p12.1 | 55566108 | G | 0.0643 |
| rs116685461 | 6p21.33 | 31315512 | G | 0.0655 |
| rs9271695 | 6p21.32 | 32593080 | G | 0.0889 |
| rs2516420 | 6p21.33 | 31449620 | C | 0.1091 |
| rs116353863 | 6p21.33 | 31010185 | C | 0.1202 |
| rs16878812 | 6p21.31 | 35569562 | A | 0.0778 |
| rs9470361 | 6p21.2 | 36623379 | A | 0.054 |
| rs62404966 | 6p12.1 | 55712124 | C | 0.0724 |
| rs3131043 | 6p21.33 | 30758466 | G | 0.0294 |
| rs2070699 | 6p24.1 | 12292772 | T | 0.0294 |
| rs1476570 | 6p22.1 | 29809860 | A | 0.0492 |
| rs3830041 | 6p21.32 | 32191339 | T | 0.0645 |
| rs6928864 | 6q21 | 105966894 | C | 0.0531 |
| rs62396735 | 6p21.1 | 41702582 | C | 0.033 |
| rs12672022 | 7p13 | 45136423 | T | 0.0067 |
| rs80077929 | 7p12.3 | 46094089 | T | 0.0093 |
| rs10951878 | 7p12.3 | 46926695 | C | 0.0531 |
| rs3801081 | 7p12.3 | 47511161 | G | 0.0253 |
| rs7013278 | 8q24.21 | 128414892 | T | 0.0091 |
| rs4313119 | 8q24.21 | 128571855 | G | 0.0518 |
| rs16892766 | 8q23.3 | 117630683 | C | 0.2099 |
| rs6469654 | 8q23.3 | 117632965 | G | 0.0677 |
| rs117079142 | 8q24.11 | 117790914 | A | 0.1139 |
| rs6983267 | 8q24.21 | 128413305 | G | 0.1052 |
| rs34405347 | 9q22.33 | 101679752 | T | 0.0089 |
| rs1537372 | 9p21.3 | 22103183 | G | 0.012 |
| rs10980628 | 9q31.3 | 113671403 | C | 0.0511 |
| rs12217641 | 10p14 | 8663875 | C | 0.0069 |
| rs10786560 | 10q24.2 | 101315166 | G | 0.0082 |
| rs1250567 | 10q22.3 | 81046265 | C | 0.047 |
| rs11255841 | 10p14 | 8739580 | T | 0.1064 |
| rs10821907 | 10q11.23 | 52648454 | C | 0.073 |
| rs704017 | 10q22.3 | 80819132 | G | 0.0765 |
| rs11190164 | 10q24.2 | 101351704 | G | 0.0889 |
| rs12246635 | 10q25.2 | 114288619 | C | 0.0975 |
| rs11196170 | 10q25.2 | 114722621 | A | 0.0527 |
| rs7946853 | 11q13.4 | 74409077 | C | 0.0119 |
| rs55864876 | 11q22.1 | 100717136 | G | 0.015 |
| rs2186607 | 11q22.1 | 101656397 | T | 0.0483 |
| rs61389091 | 11q13.4 | 74427921 | C | 0.1934 |
| rs4450168 | 11p15.4 | 10286755 | C | 0.0413 |
| rs174533 | 11q12.2 | 61549025 | G | 0.0636 |
| rs7121958 | 11q13.4 | 74280012 | G | 0.078 |
| rs3087967 | 11q23.1 | 111156836 | T | 0.1122 |
| rs4759277 | 12q13.3 | 57533690 | A | 0.0285 |
| rs1427760 | 12q24.21 | 115100714 | C | 0.0424 |
| rs3217874 | 12p13.32 | 4400808 | T | 0.0453 |
| rs10849433 | 12p13.31 | 6406904 | C | 0.0468 |
| rs11610543 | 12q12 | 43134191 | G | 0.0474 |
| rs35808169 | 12p13.32 | 4368607 | C | 0.089 |
| rs3217810 | 12p13.32 | 4388271 | T | 0.1181 |
| rs2250430 | 12p13.31 | 6421174 | T | 0.0597 |
| rs77969132 | 12p11.21 | 31594813 | T | 0.1583 |
| rs12372718 | 12q13.12 | 51171090 | G | 0.0896 |
| rs597808 | 12q24.12 | 111973358 | G | 0.0737 |
| rs7300312 | 12q24.21 | 115890922 | C | 0.066 |
| rs2710310 | 12p13.2 | 12035649 | C | 0.0145 |
| rs78341008 | 13q22.1 | 73791554 | C | 0.0109 |
| rs8000189 | 13q34 | 111075881 | T | 0.0473 |
| rs45597035 | 13q22.1 | 73649152 | A | 0.0495 |
| rs1924816 | 13q22.1 | 73997961 | A | 0.0506 |
| rs7333607 | 13q13.3 | 37462010 | G | 0.0758 |
| rs1330889 | 13q22.3 | 78609615 | C | 0.0453 |
| rs1951864 | 14q22.2 | 54369299 | A | 0.0059 |
| rs17094983 | 14q23.1 | 59189361 | G | 0.0062 |
| rs8020436 | 14q23.1 | 59208437 | A | 0.0294 |
| rs35107139 | 14q22.2 | 54419106 | C | 0.0912 |
| rs4901473 | 14q22.2 | 54445157 | G | 0.0465 |
| rs745213 | 15q23 | 68060389 | G | 0.0072 |
| rs12594720 | 15q22.31 | 67007018 | C | 0.0246 |
| rs56324967 | 15q22.33 | 67402824 | C | 0.0689 |
| rs17816465 | 15q13.3 | 33156386 | A | 0.069 |
| rs12708491 | 15q13.3 | 32992836 | G | 0.0464 |
| rs2293581 | 15q13.3 | 33010736 | A | 0.1248 |
| rs7495132 | 15q26.1 | 91172901 | T | 0.0453 |
| rs9930005 | 16q23.2 | 80043258 | C | 0.0061 |
| rs12447408 | 16q24.1 | 86252544 | A | 0.0079 |
| rs9924886 | 16q22.1 | 68743939 | A | 0.055 |
| rs12149163 | 16q24.1 | 86339315 | T | 0.0487 |
| rs62042090 | 16q24.1 | 86703949 | T | 0.0481 |
| rs983318 | 17q24.3 | 70413253 | A | 0.0397 |
| rs73975586 | 17p13.3 | 814243 | A | 0.0497 |
| rs1078643 | 17p12 | 10707241 | A | 0.0747 |
| rs75954926 | 17q25.3 | 81061048 | G | 0.0882 |
| rs373585858 | 17q25.3 | 80394556 | A | 0.1103 |
| rs4968127 | 17p13.3 | 809643 | G | 0.0514 |
| rs11874392 | 18q21.1 | 46453156 | A | 0.1606 |
| rs73068325 | 19q13.43 | 59079096 | T | 0.0066 |
| rs34797592 | 19p13.11 | 16417198 | T | 0.0824 |
| rs28840750 | 19q13.11 | 33519927 | T | 0.1939 |
| rs1963413 | 19q13.2 | 41871573 | A | 0.0441 |
| rs12979278 | 19q13.33 | 49218602 | T | 0.0293 |
| rs2738783 | 20q13.33 | 62308612 | T | 0.006 |
| rs6067417 | 20q13.13 | 48983697 | C | 0.0331 |
| rs6031311 | 20q13.12 | 42666475 | T | 0.0362 |
| rs6091189 | 20q13.13 | 49256285 | T | 0.0549 |
| rs994308 | 20p12.3 | 6603622 | C | 0.0626 |
| rs28488 | 20p12.3 | 6762221 | T | 0.0714 |
| rs556532366 | 20p12.3 | 8568071 | T | 0.0715 |
| rs189583 | 20p12.3 | 6376457 | G | 0.0795 |
| rs4813802 | 20p12.3 | 6699595 | G | 0.0819 |
| rs11087784 | 20p12.3 | 7740976 | G | 0.0874 |
| rs6066825 | 20q13.13 | 47340117 | A | 0.0719 |
| rs6063514 | 20q13.13 | 49055318 | C | 0.0547 |
| rs13831 | 20q13.32 | 57475191 | G | 0.0334 |
| rs1741640 | 20q13.33 | 60932414 | C | 0.1146 |
| rs6058093 | 20q11.22 | 33213196 | C | 0.045 |

A adenine, C cytosine, G guanine, T thymine

### **Table S2.** Baseline characteristics of study participants stratified by training or testing set in the UK Biobank cohort.

|  | **UK Biobank training set** | | | | **UK Biobank testing set** | | | |  |
| --- | --- | --- | --- | --- | --- | --- | --- | --- | --- |
| **Baseline Characteristics** | **Participants**  (n = 108,425) | **Cases**  (n = 1,337) | **Non-cases**  (n = 107,088) | ***p* value**^a^ | **Participants**  (n = 46,467) | **Cases**  (n = 542) | **Non-cases**  (n = 45,925) | ***p* value**^a^ | ***p* value**^b^ |
| **Sex,** No. (%) |  |  |  | <0.001 |  |  |  | <0.001 | 0.241 |
| Male | 48,161 (44.4) | 719 (53.8) | 47,445 (44.3) |  | 20,791 (44.7) | 321 (59.2) | 20,470 (44.6) |  |  |
| Female | 60,264 (55.6) | 618 (46.2) | 59,646 (55.7) |  | 25,676 (55.3) | 221 (40.7) | 25,455 (55.4) |  |  |
| **Age** at blood collection, in years |  |  |  | <0.001 |  |  |  | <0.001 | 0.870 |
| Mean (SD) | 54.51 (8.0) | 59.06 (7.2) | 54.45 (8.0) |  | 54.50 (7.97) | 59.27 (7.16) | 54.44 (8.0) |  |  |
| **BMI** (kg/m^2^) |  |  |  | <0.001 |  |  |  | <0.001 | 0.942 |
| Mean (SD) | 27.35 (4.82) | 27.95 (4.9) | 27.35 (4.8) |  | 27.35 (4.8) | 28.26 (4.96) | 27.34 (4.8) |  |  |
| Unknown | 397 | 4 | 393 |  | 170 | - | 170 |  |  |
| **Smoking status,** No. (%) |  |  |  | <0.001 |  |  |  | <0.001 | 0.813 |
| Never | 61,626 (56.8) | 638 (47.7) | 60,988 (57.0) |  | 26,419 (56.9) | 249 (45.9) | 26,170 (57.0) |  |  |
| Former | 34,786 (32.1) | 536 (40.1) | 34,250 (32.0) |  | 14,853 (32.0) | 240 (44.3) | 14,613 (31.8) |  |  |
| Current | 11,983 (11.1) | 163 (12.2) | 11,820 (11.0) |  | 5,180 (11.1) | 53 (9.8) | 5,127 (11.2) |  |  |
| Unknown | 30 | - | 30 |  | 15 | - | 15 |  |  |
| **Alcohol** consumption, No. (%) |  |  |  | <0.001 |  |  |  | 0.690 | 0.233 |
| Abstainer | 33,511 (30.9) | 359 (26.9) | 33,152 (31.0) |  | 14,573 (31.4) | 158 (29.2) | 14,415 (31.4) |  |  |
| Low | 43,368 (40.0) | 537 (40.2) | 42,831 (40.0) |  | 18,359 (39.5) | 223 (41.1) | 18,136 (39.5) |  |  |
| Medium | 18,600 (17.2) | 238 (17.8) | 18,362 (17.1) |  | 7,954 (17.1) | 92 (17.0) | 7,862 (17.1) |  |  |
| High | 12,946 (11.9) | 203 (15.2) | 12,743 (11.9) |  | 5,581 (12.0) | 69 (12.7) | 5,512 (12.0) |  |  |
| Unknown | - | - | - |  | - | - | - |  |  |
| **Follow-up time to CRC diagnosis**, in years |  |  |  | - |  |  |  | - |  |
| Median (Range) | - | 7.16 (0-15) | - |  | - | 6.71 (0-15) | - |  | 0.017 |

p-values were obtained from t-test for continuous variables and from chi-squared test for categorical variables. P-values indicate comparisons between cases and non-cases within the training and testing sets (ᵃ) and between the overall training and testing sets (ᵇ).

CRC colorectal cancer, No. Number, SD standard deviation

# **Table S3**. β coefficient of adjusted HR (95% CI) and *p* values of colorectal cancer for the selected metabolites in Cox proportional hazards model

| **Metabolites** | **UKB Testing set**  (n = 46,467) | | **ESTHER cohort**  (n = 3,242) | |
| --- | --- | --- | --- | --- |
|  | HR (95% CI) | *p* value | HR (95% CI) | *p* value |
| **Amino acids** |  |  |  |  |
| Total concentration of branched-chain amino acids (leucine + isoleucine + valine) | 1.06 (0.98-1.15) | 0.274 | 0.85 (0.70-1.04) | 0.283 |
| Alanine | 1.06 (0.98-1.15) | 0.274 | 1.12 (0.93-1.35) | 0.384 |
| Glutamine | 1.06 (0.97-1.15) | 0.274 | 0.65 (0.53-0.79) | **< 0.001** |
| Tyrosine | 1.01 (0.92-1.09) | 0.900 | 0.92 (0.76-1.13) | 0.647 |
| Histidine | 0.98 (0.89-1.06) | 0.647 | 0.78 (0.64-0.96) | 0.071 |
| Glycine | 0.91 (0.83-0.99) | 0.058 | 0.75 (0.61-0.93) | **0.040** |
| **Fatty acid ratios** |  |  |  |  |
| Ratio of saturated fatty acids to total fatty acids | 1.05 (0.96-1.14) | 0.396 | 1.17 (0.97-1.41) | 0.243 |
| Ratio of linoleic acid to total fatty acids | 0.89 (0.82-0.97) | **0.019** | 0.88 (0.73-1.05) | 0.329 |
| **Fluid balance** |  |  |  |  |
| Creatinine | 1.14 (1.10-1.18) | **< 0.001** | 0.87 (0.71-1.06) | 0.329 |
| Albumin | 0.85 (0.78-0.92) | **< 0.001** | 0.71 (0.59-0.85) | **0.002** |
| **Glycolysis related metabolites** |  |  |  |  |
| Glucose | 1.16 (1.08-1.25) | **< 0.001** | 0.98 (0.81-1.19) | 0.917 |
| Citrate | 1.06 (0.98-1.15) | 0.274 | 0.84 (0.70-1.01) | 0.184 |
| **Ketone bodies** |  |  |  |  |
| 3-Hydroxybutyrate | 1.11 (1.04-1.18) | **0.007** | 0.92 (0.74-1.15) | 0.647 |
| Acetate | 1.02 (0.96-1.09) | 0.587 | 0.56 (0.39-0.78) | **0.007** |
| **Lipoprotein particle sizes** |  |  |  |  |
| Average diameter for LDL particles | 0.91 (0.84-0.98) | 0.051 | 0.99 (0.82-1.20) | 0.917 |
| **Lipoprotein subclasses** |  |  |  |  |
| Phospholipids in small LDL | 1.10 (1.01-1.20) | 0.052 | 1.01 (0.84-1.23) | 0.917 |
| Concentration of small HDL particles | 1.01 (0.93-1.10) | 0.900 | 0.72 (0.59-0.88) | **0.008** |
| **Relative lipoprotein lipid concentrations** |  |  |  |  |
| Triglycerides to total lipids ratio in very small VLDL | 1.16 (1.07-1.26) | **0.002** | 0.97 (0.80-1.17) | 0.827 |
| Cholesterol to total lipids ratio in very large HDL | 1.12 (1.02-1.24) | 0.052 | 0.97 (0.83-1.13) | 0.822 |
| Cholesteryl esters to total lipids ratio in chylomicrons and extremely large VLDL | 1.05 (0.96-1.15) | 0.344 | 0.96 (0.79-1.16) | 0.819 |
| Free cholesterol to total lipids ratio in large VLDL | 1.04 (0.95-1.14) | 0.492 | 1.20 (0.85-1.70) | 0.513 |
| Phospholipids to total lipids ratio in small LDL | 0.99 (0.91-1.08) | 0.900 | 1.08 (0.88-1.31) | 0.647 |
| Cholesteryl esters to total lipids ratio in IDL | 0.90 (0.83-0.98) | **0.036** | 0.84 (0.71-0.99) | 0.102 |

Cause-specific Cox proportional hazards models were used to estimate hazard ratios (HR) and 95% confidence intervals (CI) for the association between selected metabolites and CRC risk. HRs were reported per 1-SD increase of the log1p-transformed value for each metabolite. Multiple testing correction was applied using the Benjamini-Hochberg method. P-values set in boldface indicate statistical significance.

CI confidence interval, HDL high-density lipoproteins, HR hazard ratio, IDL intermediate-density lipoproteins, LDL low-density lipoproteins, VLDL very low-density lipoproteins

**Table S4**. Performance of the individual and combined risk prediction panels (5-year and 10-year follow-up), by internal and external validation

|  | **5-year-follow-up** | | **10-year-follow-up** | |
| --- | --- | --- | --- | --- |
|  | **Internal validation**  (UKB Testing set, n = 46,467, CRC cases = 201) | **External validation**  (ESTHER cohort, n = 3,242, CRC cases = 38 ) | **Internal validation**  (UKB Testing set, n = 46,467,  CRC cases = 447) | **External validation**  (ESTHER cohort, n = 3,242,  CRC cases = 64) |
|  | C-Index (95% CI) | | C-Index (95% CI) | |
| **Individual panels** | | | | |
| Metabolomics Risk Panel (MRP) | 0.590 (0.551, 0.624) | 0.565 (0.471, 0.655) | 0.603 (0.578, 0.630) | 0.551 (0.483, 0.617) |
| Environmental Risk Panel (ERP) | 0.709 (0.670, 0.746) | 0.718 (0.620, 0.763) | 0.686 (0.661, 0.709) | 0.687 (0.626, 0.745) |
| Genetic Risk Panel (GRP) | 0.647 (0.608, 0.683) | 0.613 (0.526, 0.710) | 0.640 (0.615, 0.665) | 0.634 (0.568, 0.704) |
| **Combined panels** | | | | |
| MRP + GRP | 0.662 (0.624, 0.695) | 0.628 (0.537, 0.727) | 0.670 (0.646, 0.694) | 0.638 (0.570, 0.710) |
| MRP + ERP | 0.703 (0.665, 0.739) | 0.727 (0.628, 0.805) | 0.686 (0.664, 0.710) | 0.680 (0.617, 0.739) |
| GRP + ERP | 0.742 (0.705, 0.777) | 0.737 (0.660, 0.806) | 0.727 (0.703, 0.749) | 0.727 (0.669, 0.782) |
| MRP + GRP + ERP | 0.738 (0.701, 0.772) | 0.739 (0.654, 0.820) | 0.726 (0.705, 0.749) | 0.746 (0.658, 0.771) |

Model performance was assessed using the concordance index (C-index), with 95% confidence intervals obtained through bootstrap resampling (1,000 iterations). The UKB training set was used to develop the models, which were internally validated in the UKB testing set and externally validated in the ESTHER cohort. Results are presented for 5-year follow-up and 10-year follow-up periods.

CI confidence interval, CRC colorectal cancer, UKB UK Biobank

**Table S5**. Diagnostic performance of NRI and IDI between MRP and the combined models

|  | **GRP vs. MRP + GRP** | **ERP vs. MRP + ERP** | **GRP + ERP vs. MRP + GRP + ERP** |
| --- | --- | --- | --- |
| **Internal validation** (UKB Testing set, n = 46,467) | | | |
| NRI (95% CI) | 0.189 (0.120, 0.258) | -0.005 (-0.016, 0.005) | -0.005 (-0.015, 0.005) |
| NRI for events (95% CI) | -0.576 (-0.644, -0.507) | 0.992 (0.982, 1.003) | 0.993 (0.982, 1.003) |
| NRI for non-events (95% CI) | 0.764 (0.758, 0.770) | -0.998 (-0.999, -0.997) | -0.997 (-0.998, -0.996) |
| IDI | 0.045 (0.036, 0.053) | 0.004 (0.003, 0.004) | 0.005 (0.004, 0.006) |
| **External validation** (ESTHER cohort, n = 3,242) | | | |
| NRI (95% CI) | 0.022 (-0.099, 0.144) | 0.005 (0.002, 0.009) | -0.009 (-0.047, 0.029) |
| NRI for events (95% CI) | -0.786 (-0.906, -0.667) | 1.000 (1.000, 1.000) | 0.981 (0.943, 1.018) |
| NRI for non-events (95% CI) | 0.809 (0.788, 0.830) | -0.994 (-0.998, -0.991) | -0.990 (-0.995, -0.985) |
| IDI | 0.021 (0.002, 0.040) | 0.003 (0.001, 0.005) | 0.004 (0.002, 0.006) |

The net reclassification index (NRI) and integrated discrimination improvement (IDI) were used to evaluate whether incorporating the metabolomics risk panel into the genetic risk panel, environmental risk panel, or their combination improved model performance.

CI confidence interval, ERP environmental risk panel, GRP genetic risk panel, IDI integrated discrimination improvement index, MRP metabolomics risk panel, NRI net reclassification index, UKB UK Biobank.

**Table S6**. Discriminatory ability of the MRP in subgroups defined by ERP risk factors

|  | **Internal validation**  (UKB Testing set, n = 46,467) | | **External validation**  (ESTHER cohort, n = 3,242) | |
| --- | --- | --- | --- | --- |
|  | *n* Cases | C-index (95% CI) | *n* Cases | C-index (95% CI) |
| **Sex** |  |  |  |  |
| Male | 321 | 0.596 (0.565, 0.625) | 68 | 0.565 (0.494, 0.634) |
| Female | 221 | 0.595 (0.559, 0.634) | 35 | 0.466 (0.371, 0.566) |
| **Age** at blood collection |  |  |  |  |
| < 60 y | 242 | 0.562 (0.526, 0.602) | 22 | 0.610 (0.496, 0.712) |
| ≥ 60 y | 300 | 0.568 (0.534, 0.600) | 81 | 0.566 (0.506, 0.628) |
| **BMI** (kg/m^2^) |  |  |  |  |
| < 25 | 137 | 0.586 (0.538, 0.638) | 16 | 0.553 (0.408, 0.678) |
| 25-30 | 246 | 0.562 (0.525, 0.598) | 56 | 0.541 (0.471, 0.614) |
| > 30 | 159 | 0.602 (0.557, 0.647) | 31 | 0.440 (0.340, 0.539) |
| **Smoking status** |  |  |  |  |
| Never | 249 | 0.585 (0.548, 0.624) | 45 | 0.496 (0.406, 0.585) |
| Former | 240 | 0.589 (0.554, 0.623) | 47 | 0.640 (0.556, 0.710) |
| Current | 53 | 0.605 (0.521, 0.685) | 11 | 0.570 (0.392, 0.743) |
| **Alcohol consumption** |  |  |  |  |
| Abstainer/Low | 381 | 0.606 (0.576, 0.635) | 96 | 0.516 (0.450, 0.576) |
| Medium/High | 161 | 0.606 (0.562, 0.648) | 7 | 0.721 (0.523, 0.895) |

Model performance was assessed using the concordance index (C-index), with 95% confidence intervals obtained through bootstrap resampling (1,000 iterations). Performance was assessed in subgroups defined by environmental risk panel (ERP) risk factors to evaluate potential interactions between environmental risk factors and the metabolomics risk panel (MRP).

BMI body mass index, CI confidence interval, ERP environmental risk panel, MRP metabolomics risk panel, UKB UK Biobank, y year

|  | **Internal validation**  (UKB Testing set, n = 46,467) | | | | **External validation**  (ESTHER cohort, n = 3,242) | | | |
| --- | --- | --- | --- | --- | --- | --- | --- | --- |
|  | *n* Cases | HR (95% CI) | *p* value | *p* ^interaction^ | *n* Cases | HR (95% CI) | *p* value | *p* ^interaction^ |
| **Sex** | | | | | | | | |
| Male | 321 | 1.25 (1.15, 1.37) | **< 0.001** | 0.598 | 68 | 1.48 (0.86, 2.55) | 0.447 | 0.393 |
| Female | 221 | 1.29 (1.16, 1.43) | **< 0.001** |  | 35 | 0.94 (0.34, 2.64) | 0.913 |  |
| **Age** at blood collection | | | | | | | | |
| < 60 y | 242 | 1.29 (1.16, 1.44) | **< 0.001** | 0.469 | 22 | 2.07 (0.70, 6.07) | 0.447 | 0.285 |
| ≥ 60 y | 300 | 1.18 (1.05, 1.34) | **0.008** |  | 81 | 1.19 (0.67, 2.10) | 0.902 |  |
| **BMI** (kg/m^2^) | | | | | | | | |
| < 25 | 137 | 1.25 (1.11, 1.41) | **< 0.001** | 0.875 | 16 | 0.82 (0.16, 4.19) | 0.913 | 0.921 |
| 25-30 | 246 | 1.24 (1.10, 1.42) | **< 0.001** |  | 56 | 1.60 (0.89, 2.88) | 0.447 |  |
| > 30 | 159 | 1.44 (1.23, 1.68) | **< 0.001** |  | 31 | 1.10 (0.41, 2.98) | 0.913 |  |
| **Smoking status** | | | | | | | | |
| Never | 249 | 1.66 (1.37, 2.01) | **< 0.001** | 0.065 | 45 | 0.78 (0.31, 1.96) | 0.902 | 0.203 |
| Former | 240 | 1.21 (1.10, 1.33) | **< 0.001** |  | 47 | 1.92 (1.11, 3.34) | 0.242 |  |
| Current | 53 | 1.79 (1.21, 2.63) | **0.004** |  | 11 | 1.40 (0.25, 7.82) | 0.913 |  |
| **Alcohol consumption** | | | | | | | | |
| Abstainer/Low | 381 | 1.26 (1.17, 1.36) | **< 0.001** | **0.001** | 96 | 1.44 (0.86, 2.40) | 0.447 | 0.834 |
| Medium/High | 161 | 1.73 (1.39, 2.17) | **< 0.001** |  | 7 | 1.84 (0.47, 7.17) | 0.761 |  |

**Table S7**. Cox proportional hazards models of the MRP in subgroups defined by ERP risk factors

Cause-specific Cox proportional hazards models were used to estimate hazard ratios (HR) and 95% confidence intervals for the association between MRP and CRC risk, stratified by risk factors included in the environmental risk panel (ERP), namely sex, age, BMI, smoking status, and alcohol consumption. Additionally, multiplicative interaction terms (MRP × Risk Factor) were introduced into the models to assess whether the association between MRP and CRC risk varied across these subgroups. HRs were reported per 1-SD increase of the MRP. Multiple testing correction was applied using the Benjamini-Hochberg method, and p-values set in boldface indicate statistical significance.

BMI body mass index, CI confidence interval, CRC colorectal cancer, HR Hazard Ratio, UKB UK Biobank, y year

**Figure S1.** Correlation matrix of the selected metabolites in the UK Biobank training set

**B**. After exclusion of highly correlated metabolites

**A**. Before exclusion of highly correlated metabolites


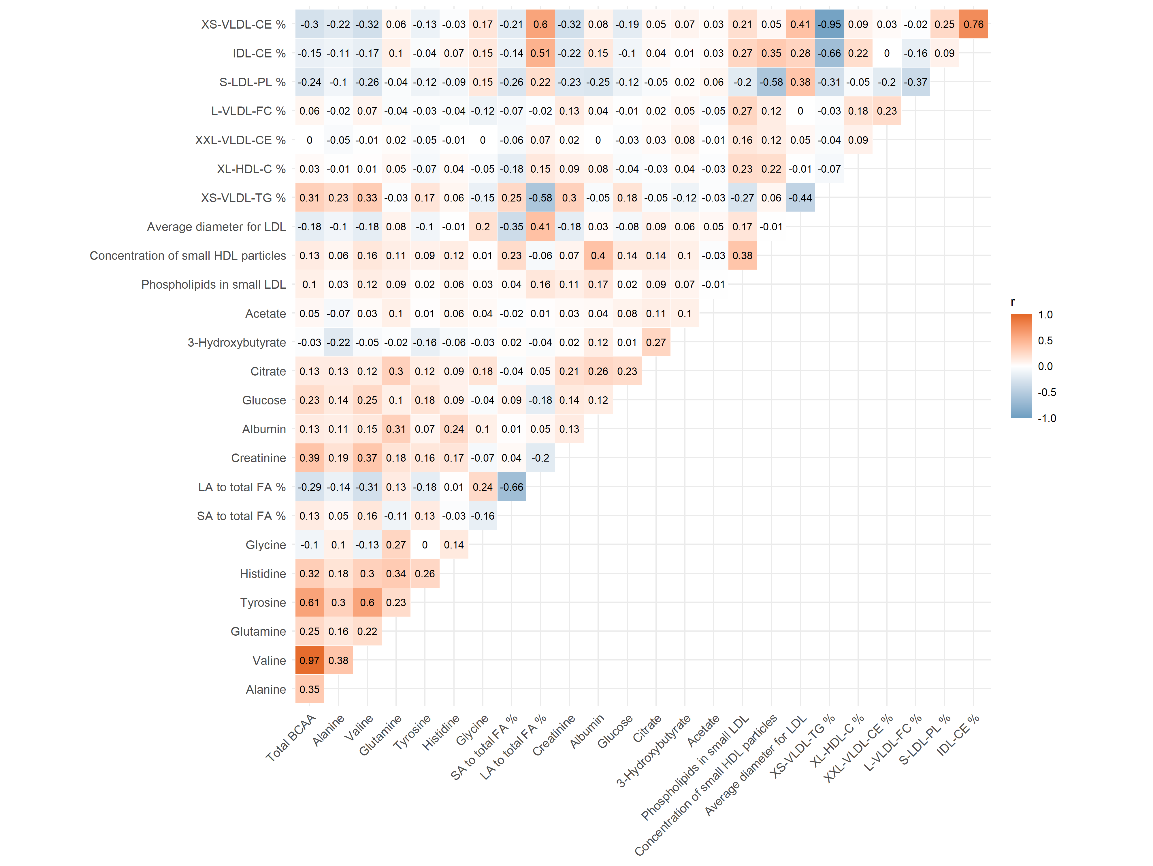

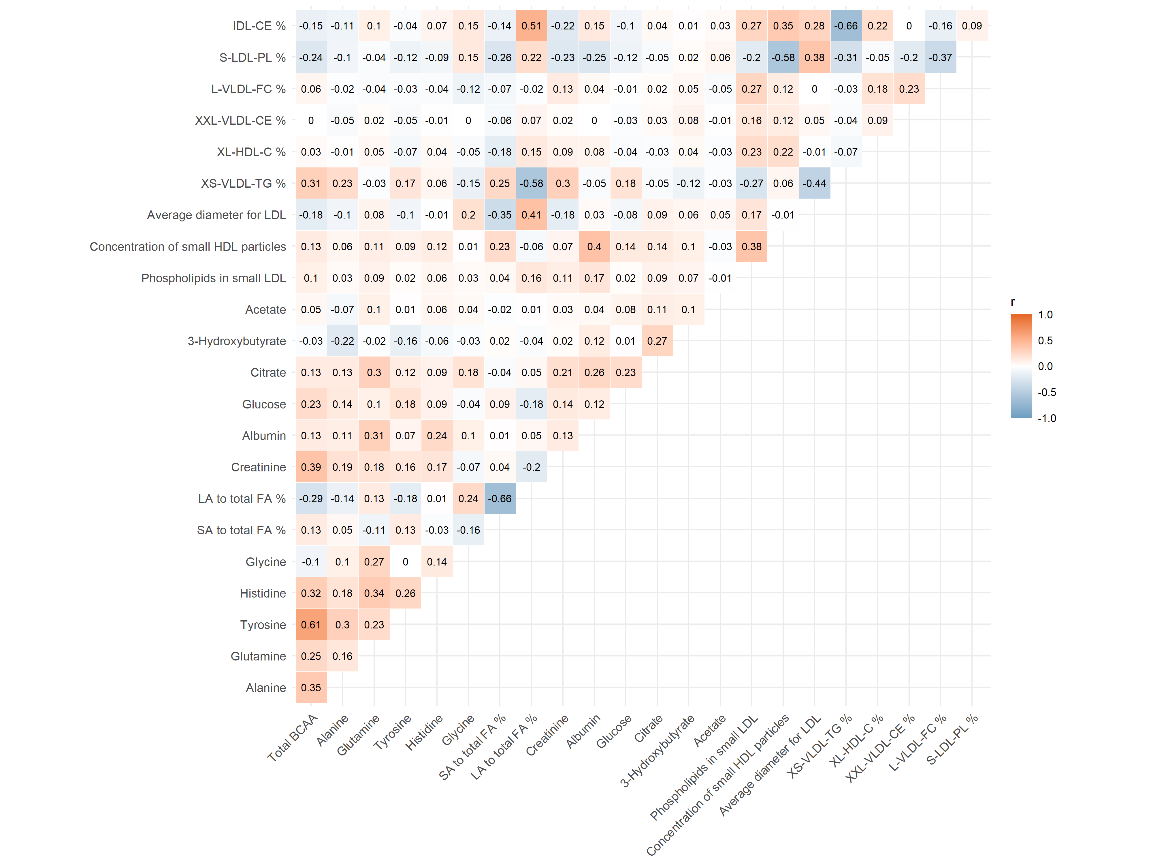


Heatmaps of Pearson correlation coefficients were generated to visualize collinearity among the selected metabolites in the metabolomics risk panel (MRP), both before and after excluding the highly correlated metabolites valine and the cholesteryl esters to total lipids ratio in very small VLDL.

BCAA branched-chain amino acids, C cholesterol, CE cholesteryl esters, CI confidence interval, FA fatty acids, FB fluid balance, FC free cholesterol, Glycolysis glycolysis-related metabolites, HDL high-density lipoproteins, HR hazard ratio, Ketone ketone bodies, L large, LA linoleic acid, LDL low-density lipoproteins, M medium, PL phospholipids, S small, TG triglycerides, UKB UK Biobank, VLDL very low-density lipoproteins, XL very large, XS very small, XXL extremely large, % ratio.
